# Federated and Transfer Learning Methods for the Classification of Melanoma and Nonmelanoma Skin Cancers: A Prospective Study

**DOI:** 10.3390/s23208457

**Published:** 2023-10-13

**Authors:** Shafia Riaz, Ahmad Naeem, Hassaan Malik, Rizwan Ali Naqvi, Woong-Kee Loh

**Affiliations:** 1 Department of Computer Science, National College of Business Administration & Economics Sub Campus Multan, Multan 60000, Pakistan; shahfiariaz@gmail.com (S.R.); f2019288004@umt.edu.pk (H.M.); 2 Department of Computer Science, University of Management and Technology, Lahore 54000, Pakistan; f2019288007@umt.edu.pk; 3 Department of Intelligent Mechatronics Engineering, Sejong University, Seoul 05006, Republic of Korea; 4 School of Computing, Gachon University, Seongnam 13120, Republic of Korea

**Keywords:** transfer learning, federated learning, melanoma, dermoscopy, skin cancer

## Abstract

Skin cancer is considered a dangerous type of cancer with a high global mortality rate. Manual skin cancer diagnosis is a challenging and time-consuming method due to the complexity of the disease. Recently, deep learning and transfer learning have been the most effective methods for diagnosing this deadly cancer. To aid dermatologists and other healthcare professionals in classifying images into melanoma and nonmelanoma cancer and enabling the treatment of patients at an early stage, this systematic literature review (SLR) presents various federated learning (FL) and transfer learning (TL) techniques that have been widely applied. This study explores the FL and TL classifiers by evaluating them in terms of the performance metrics reported in research studies, which include true positive rate (TPR), true negative rate (TNR), area under the curve (AUC), and accuracy (ACC). This study was assembled and systemized by reviewing well-reputed studies published in eminent fora between January 2018 and July 2023. The existing literature was compiled through a systematic search of seven well-reputed databases. A total of 86 articles were included in this SLR. This SLR contains the most recent research on FL and TL algorithms for classifying malignant skin cancer. In addition, a taxonomy is presented that summarizes the many malignant and non-malignant cancer classes. The results of this SLR highlight the limitations and challenges of recent research. Consequently, the future direction of work and opportunities for interested researchers are established that help them in the automated classification of melanoma and nonmelanoma skin cancers.

## 1. Introduction

Skin cancer is the most common type of cancer. Clinical screenings are performed first; then, biopsy, histological tests, and dermoscopy are performed to confirm the diagnosis [1]. Skin cancer appears when the normal growth of skin cells is affected, causing a mutation in the DNA and eventually leading to skin cancer. Exposure to ultraviolet rays is considered to be the main cause of skin cancer. However, several other factors, such as a light skin tone, exposure to radiation and chemicals, severe skin injuries/burns, a weak immune system, old age, and smoking, also lead to skin cancer [2]. According to data compiled by the WHO, cancer is the main cause of death globally. They reveal that cancer cases are increasing rapidly, with one in six deaths occurring due to this deadly disease. In 2018, 18.1 million people had cancer globally, and approximately 9.6 million died from this disease. It is predicted that these statistics will nearly double by 2040 and approximately 29.4 million people will be diagnosed with cancer [3]. The most frequently diagnosed cancers worldwide are stomach, lung, liver, skin, cervix, breast, and colorectal [4]. This disease is the most severe and critical issue in all generations of populations, regardless of social status or wealth. At an early stage, the treatment and diagnosis of cancer can significantly decrease the number of deaths [5]. Researchers are mainly concerned with diagnosing cancer early by employing artificial intelligence-based approaches [6]. There are several classes of skin cancer that are considered nonmelanoma cancers. Basal cell carcinoma (BCC), Merkel cell carcinoma (MCC), and squamous cell carcinoma (SCC) are examples of nonmelanoma skin malignancies. These nonmelanoma cancers are considered to be less aggressive than melanoma. Furthermore, these nonmelanoma cancers are more treatable than melanoma [7].

The most malignant type of skin cancer is melanoma, which has a high misdiagnosis rate due to its potential for metastasis, recurrence, and vascular invasion [8]. It is the 19th most common cancer among human beings. In 2018, approximately 300,000 new cases of the disease were found. Moreover, 4740 males and 2490 females died from melanoma in 2019 [9]. A report issued by the American Cancer Society in 2022 calculated that about 99,780 people will be infected with melanoma in the U.S. and approximately 7650 human beings are expected to die from it [10]. The actual cause of melanoma has still not been found, but various factors like environmental and genetic factors and ultraviolet radiation are the primary causes of skin cancer. Melanoma cancer originates in skin melanocytes, which make dark pigments in the hair, eyes, and skin [11]. Over the last few years, melanoma cancer cases have been gradually increasing. If the cancer is detected at the initial level, a minor surgical process can increase the possibility of recovery. The dermoscopy imaging technique is a popular non-invasive technique widely used by dermatologists to evaluate pigmented skin lesions [12]. Through dermoscopy, the structure becomes more visible for examination by the dermatologist because it enlarges the lesion’s position or surface [13,14]. However, this imaging technique can only be effective if it is used by trained and expert dermatologists because it is wholly based on the physician’s experience and optical acuteness [15]. These challenges and issues stimulate researchers to discover new strategies and methods for diagnosing and visualizing melanoma and nonmelanoma skin cancer. A computer-aided diagnosis (CAD) system, applied as a traditional process, due to its convenient and user-friendly procedure which assists young and non-experienced dermatologists in diagnosing melanoma. A proficient and experienced dermatologist can achieve 65% to 75% precision in classifying melanoma cancer through dermoscopy photographs [16]. The automated process of melanoma diagnosis from medical imaging can help dermatologists in their clinical routine. The challenges in the field of dermatology drive research groups to place their primary attention on the diagnosis of melanoma by using AI-based tools. The utilization of artificial intelligence (AI) for the diagnosis of skin cancer has recently gained a great deal of attention.

Researchers have attained many advancements using AI, mainly in finding patterns of diseases from medical imaging [17]. AI-based tools and applications in the field of dermatology are being designed to analyze the severity of psoriasis [18], and these AI-based tools involve the development of a computer algorithm that can self-learn specific dermatological tasks, such as classifying skin lesions as melanoma or nonmelanoma skin cancer [19,20]. Implementing federated learning-, deep learning-, and transfer learning-based technologies yields massive benefits for patients and dermatologists in predicting and diagnosing suspicious skin lesions. In one meta-analysis report, an AI-based tool’s diagnostic true positive rate was more significant than the dermoscopy technique (91% versus 88%) [21].

In this SLR, we pursued different federated and transfer learning algorithms, benchmark public databases, and private and non-listed datasets for melanoma classification. We conducted this SLR to provide a comprehensive literature source on transfer and federated learning [22] techniques for the diagnosis of malignant melanoma and nonmelanoma skin cancer [23,24]. Over the past few years, substantial research has been conducted on the automatic diagnosis of melanoma and other cancers by using transfer learning and deep learning techniques [23,25,26,27]. To our knowledge, no SLR is available on diagnosing melanoma disease through CNN-based pretrained models. Only one research paper was on deep learning approaches for classifying malignant melanoma skin cancer. Collecting information and assessing, summarizing, and classifying state-of-the-art models remain crucial for SLRs [28]. The primary purpose of this comprehensive SLR is to provide a state-of-the-art summary representing the scope of transfer learning [29,30] and federated learning models [31] for the detection of melanoma and nonmelanoma skin cancer, and also to demonstrate the primary inadequacy of existing approaches and fields of research where further enhancement should soon be carried out. A taxonomy diagram of melanoma and nonmelanoma skin cancer is proposed by exploring and investigating recent state-of-the-art studies. Furthermore, this study identifies the challenges, open issues, opportunities, and modern research trends for melanoma and nonmelanoma skin cancer diagnosis.

The present study is organized as follows: In Section 2, we provide a thorough description of the research method utilized to search, screen, and select the literature. Section 3 presents relevant review works conducted for diagnosing melanoma and nonmelanoma skin cancer using federated and transfer learning methods. In Section 4, we present a performance evaluation of different methods. Section 5 describes the available datasets for the diagnosis of skin cancer. Section 6 provides the results and discussion. Section 7 contains the taxonomy. In Section 8, the main findings and research gaps are discussed. Finally, in Section 9, we conclude this study.

## 2. Materials and Methods

**Figure 1:**
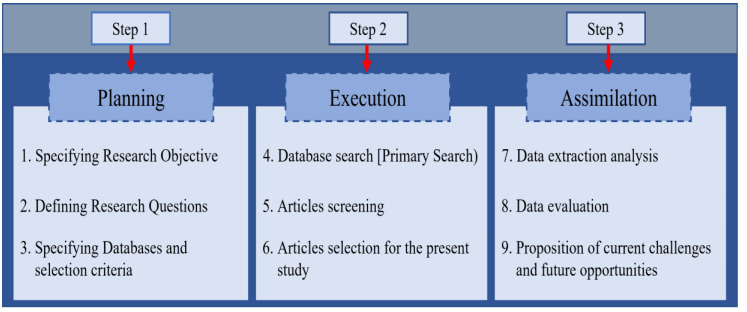
Systematic study process.

According to Petersen et al. [32], the main objective of an SLR is to provide an overview of a research area and types of research studies and identify the results available. The primary goal of an SLR is to map the number of research publications over time to identify various research trends; the secondary goal is to explore the research publication forum. The first step of this study was to set up an SLR process. This helps to identify the search strategy for published related articles. This procedure includes research objectives, research questions, keywords of the search string for the identification of the research publications, publication sources such as conferences, journals, and symposiums, and the study selection process based on exclusion and inclusion criteria. Figure 1 shows an overview of this SLR. This SLR aimed to find the techniques, federated and transfer learning classifiers, and various datasets for diagnosing melanoma and nonmelanoma skin cancer.

### 2.1. Research Objectives (ROs)

The overall objective of this SLR was to summarize and gain insight into the latest pretrained and federated learning techniques for the detection of melanoma and nonmelanoma skin cancer. The ROs of conducting this SLR were:

- To emphasize the latest research trends in TL and FL methods for detecting melanoma and nonmelanoma cancer;
- To explore the existing approaches and present an SLR of these approaches based on classification performances;
- To explore different types of available datasets for melanoma and nonmelanoma diagnosis;
- To propose a taxonomy to emphasize effective frameworks for melanoma diagnosis;
- To explore the state-of-the-art research trends, opportunities, and challenges for other researchers in diagnosing melanoma.

### 2.2. Research Questions (RQs)

This systematic mapping study aimed to summarize and gain insight into the latest pretrained and federated learning techniques for detecting melanoma and nonmelanoma skin cancer. This systematic mapping study consists of three research questions to obtain a comprehensive review of this topic. The possible answers to these research questions were extracted through the published literature, as stated in the proposed methodology by Kitchenham et al. [33]. The research questions along with the corresponding motivations are illustrated in Table 1.

### 2.3. Search Strategy

**Figure 2:**
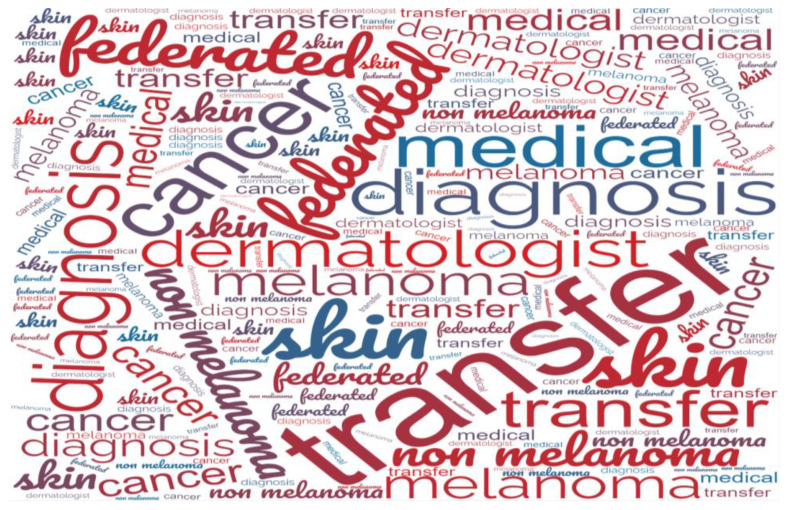
Constructed key framework.

The articles, which used TL and FL algorithms using dermoscopy images for the diagnosis of melanoma and nonmelanoma skin cancers, were identified by searching 7 different well-reputed venues: the IEEE Digital Library, the Wiley Library, Springer, the ACM Digital Library, Science Direct, Scopus, Ovid MEDLINE, and conference proceedings for articles online from January 2018 to July 2023. Manual search operations were also performed for related published articles and citations, which might have been omitted throughout the search. The amalgamation of primary, secondary, and additional keywords was used to make search strings to find related articles from databases. Moreover, “AND operators” were used for different-level keywords and “OR operators” were used for same-level keywords. The following search terms were formulated with the amalgamation of search keywords: (“transfer learning” OR “pre-trained model” OR “neural network” OR “AI” OR “artificial intelligence” OR “deep learning” OR “federated learning”) AND (“Melanoma” OR “skin lesion” OR “skin cancer” OR “non-melanoma”) AND (“detection” OR “classification” OR “diagnosis”). The retrieved results from different information sources consisted of the paper’s title, abstract, and publication source, which were further filtered according to the exclusion and inclusion selection criteria and saved in a personal knowledge base. Furthermore, a word cloud analysis of author-indexed keywords revealed that the emphasis of the articles was on “melanoma”, ”non melanoma” “cancer”, “diagnosis”, “skin”, “transfer”, “federated”, “dermatologists”, and “medical”, as graphically represented in Figure 2.

### 2.4. Study Inclusion and Exclusion Criteria

The selection procedure aimed to find and include the most important research publications on skin cancer. We only examined the article once if it appeared in several sources. The inclusion criteria of the acquired research articles were limited by the search strategies. For each selected article, we independently evaluated its eligibility by screening the titles of the search results and abstracts. After evaluating the papers that met the established inclusion criteria, our next step was to establish the exclusion criteria to omit articles that met at least one of the subsequent exclusion criteria (EC):

**EC1.**  *Research studies that were not focused on the classification of skin cancer without medical images were eliminated.*

**EC2.**  *Research studies that did not address any of the research questions presented in our SLR were eliminated.*

**EC3.**  *Research that presented a review on skin cancer was eliminated.*

**EC4.**  *Research studies that were not based on skin cancer classification were eliminated.*

### 2.5. Screening and Selection Criteria

**Figure 3:**
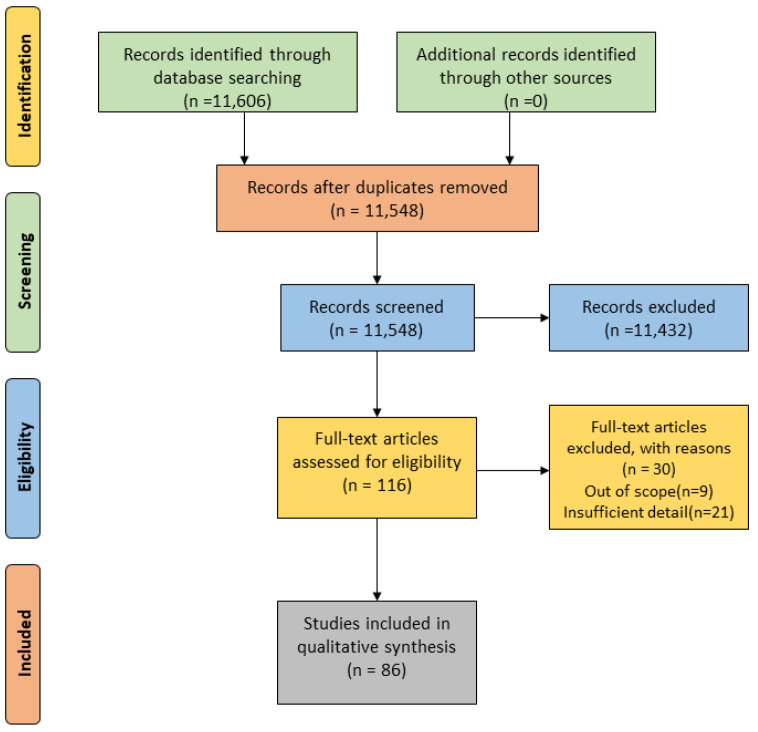
Prisma flowchart of the study selection process. Flowchart summarizes the selection and inclusion process of studies in this systematic review, including the reasons for excluding all reviewed full-text articles.

The study selection process was accomplished by finding the most related research studies. Papers that provided a significant contribution to research were selected in this systematic review. The PRISMA method was utilized in this research. Initially, 11,606 articles were identified, as shown in Figure 3. The overall search process yielded 984 articles from IEEE Xplore, 6113 from Science Direct, 239 from the ACM Digital Library, 2817 from Springer Link, 909 from Medline, 45 from Scopus, and 499 from the Wiley Online Library. Most articles in the search results were unrelated to our research questions. In the following stages, duplicate and irrelevant articles were manually excluded based on titles. A total of 11,548 articles were screened. Articles were omitted based on exclusion criteria and, finally, 86 studies were finalized and included in this SLR.

### 2.6. Search Results

**Figure 4:**
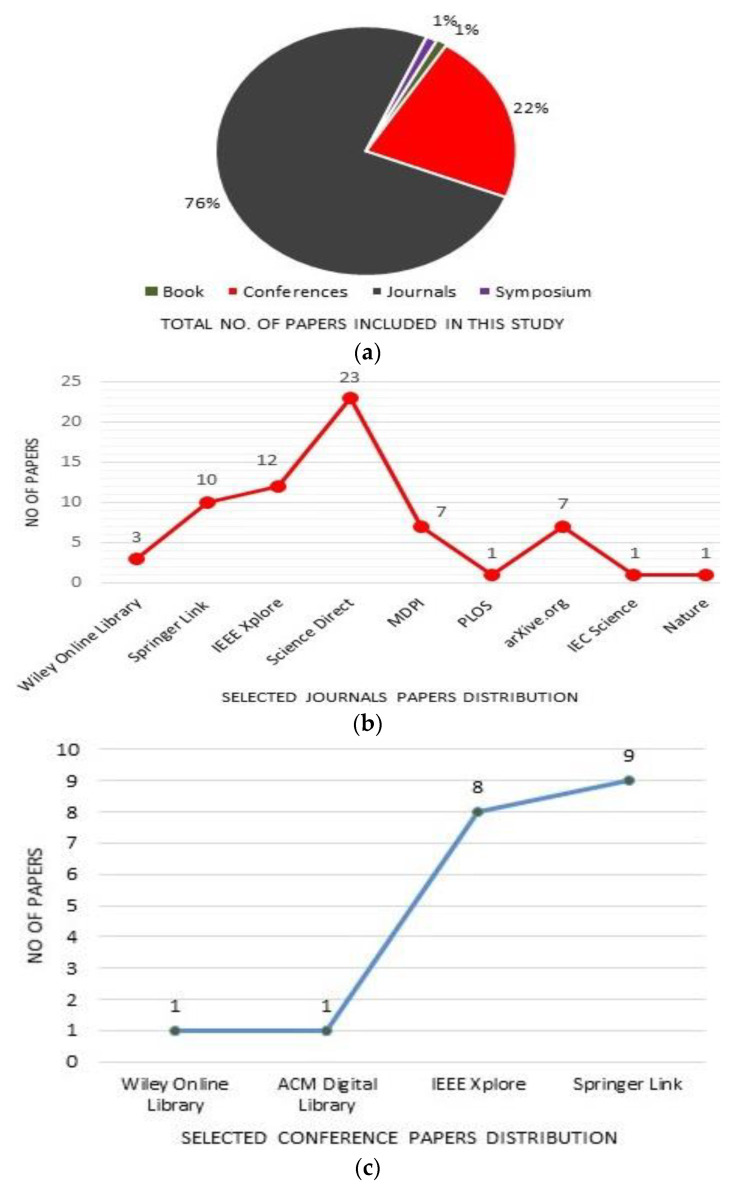
Distribution of 86 included studies: (**a**) Total no of studies included by various publication types, and included articles in this SLR from different (**b**) databases journals, and (**c**) Conference proceedings are represented.

The 86 included studies were obtained from different publications, including journals, books, symposiums, and conferences. It was computed that 76% of the selected papers were published in journals, while 1% of papers were book chapters and symposiums, respectively. However, 22% of the 86 selected studies were published at conferences. The overall distribution of all 86 included studies and the journal-wise and conference-wise distribution of the articles are presented in Figure 4.

## 3. Methods for the Detection of Melanoma and Nonmelanoma Skin Cancer (RQ1)

In the field of transfer learning and federated learning, there are several new algorithms and techniques for classifying melanoma and nonmelanoma skin cancer. In this section, state-of-the-art methods dependent on transfer learning and federated learning are examined.

### 3.1. Fully Convolutional Network (FCN)-Based Methods

Some studies used FCN-based methods to classify skin cancer, such as Lequan et al. [34], which proposed a two-stage approach for automated skin cancer recognition based on deep CNNs. FCRN and deep residual DRN networks were used for lesion segmentation and classification. The residual learning technique is utilized for the training of both deep networks. Moreover, the proposed approach creates a grade map of the skin lesion from the images and then the lesion mask is cropped and resized. The cropped lesion patch is transferred for melanoma classification. However, Al-Masni [35] proposed an integrated deep learning two-level framework for segmenting and classifying multiple skin lesions. Firstly, an FRCN is applied to dermoscopic images to segment the lesion boundaries. To differentiate between various skin lesions, the segmented skin lesions are fed into pretrained algorithms, such as DenseNet-201, Inception-ResNet-v2, ResNet-50, and Inception-v3. The pre-segmentation phase enables these classifiers to memorize and learn only specific lesion features while ignoring the surrounding region.

In comparison, Jayapriya and Jacob [36] also designed an architecture consisting of two fully convolutional networks (FCNs) based on pretrained GoogLeNet and VGG-16 models. These hybrid pretrained networks extract more specific features and give a better segmentation output than an FCRN. The segmented lesion image is next passed into a DRN and a hand-crafted feature for classification purposes. The SVM classifier is implemented for classifying various skin lesions into nonmelanoma and melanoma lesions. Elsewhere, Khan et al. [37] suggested a method for the multiclass localization and classification of lesion images based on MASK-RCNN and Resnet50 along with a feature pyramid network (FPN).

**Figure 5:**
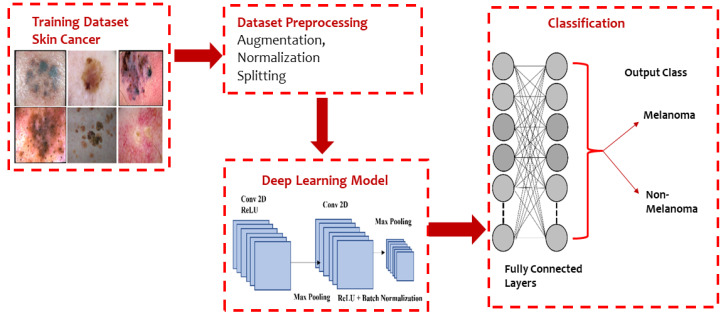
Typical CNN architecture for melanoma and non-melanoma cancer classification.

Moreover, Al-Masni et al. [38] presented an integrated model based on an FRCN and ResNet-50 network. An FRCN is implemented on dermoscopy images to segment the boundaries of the lesion images and then passed to a pretrained ResNet-50 deep residual network by fine-tuning the model for the classification of various lesion images. The basic architecture of a CNN model used for classifying melanoma and nonmelanoma is presented in Figure 5.

### 3.2. Hybrid Methods

Many studies used hybrid methods for the diagnosis of skin cancer. Kassem et al. [39] proposed an architecture that can accurately classify eight different kinds of skin lesion images, even imbalanced images between classes. The proposed method used a pretrained GoogLeNet architecture by incorporating supplementary filters onto every layer for improved feature extraction and less background noise. The model was implemented to classify the lesion by changing various layers in two ways. This change aimed to identify outliers or unknown images. The performance metrics of the architecture increased when all the layers’ weights were fine-tuned instead of performing fine-tuning only on replaced layers. Gavrilov et al. [40] used a pretrained neural network, Inception v3, that was trained on a large image dataset. Miglani et al. [41] used a novel scaling pretrained model called EfficientNet-B0 to classify lesion images in various categories by using transfer learning. Moreover, Hosny et al. [42] developed a method based on pretrained AlexNet and transfer learning to classify seven different kinds of lesions.

**Figure 6:**
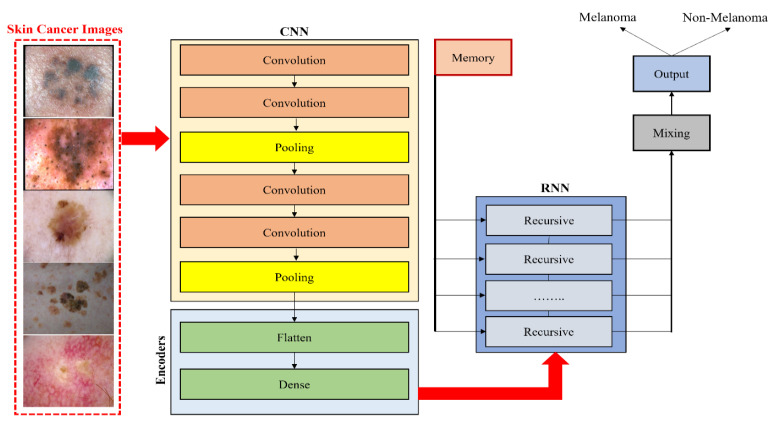
Hybrid CNN model with RNN for classifying melanoma and nonmelanoma skin disease.

Esteva et al. [43] implemented a pretrained GoogLeNet Inception v3 classifier for the binary classification of two problems: benign nevi versus malignant melanomas and benign seborrheic keratosis versus keratinocyte carcinomas. Furthermore, Majtner et al. [44] suggested a two-part method consisting of a feature extraction and feature reduction process based on deep learning with the LDA approach for melanoma detection. Pretrained AlexNet was used for feature extraction and then the LDA approach was employed to optimize features, which helped decrease the set of features and enhance the precision of classification. Ather et al. [45] proposed a multiclass classification framework for identification and optimal discrimination between different skin lesions, both benign and malignant. Moreover, three deep models, namely ResNet-18, VGG16, and AlexNet, were suggested by Mahbod et al. [46] for the classification of three lesion classes: benign nevi, malignant melanoma, and seborrheic keratosis. In comparison, Namozov et al. [47] suggested a deep method with adaptive linear piecewise (APL) activation units for the classification of melanoma that can attain superb melanoma recognition performance. Hosny et al. [48] suggested a deep CNN that classifies three different lesion types, melanoma, atypical nevus, and common nevus, from color images of skin cancer in addition to image augmentation and fine-tuning. To categorize the three types of lesions, the last layer of a pretrained AlexNet is modified. This technique can work directly with any photographic or dermoscopic image and does not need preprocessing. Devansh et al. [49] developed an automated technique for melanoma diagnosis that specially deals with skin lesion image datasets that are small-scale, extremely imbalanced, and image occlusions. However, Maron et al. [50] examined the brittleness of three pretrained VGG16, ResNet50, and DenseNet121 CNNs in image analysis and showed brittleness, such as rotation, scaling, or minor changes in the input image, which have a significant effect on the classification of the CNN. Rivera et al. [51] proposed a technique for the early detection of melanoma that is implemented on mobile phones or embedded devices. The proposed system uses a pretrained MobileNet. Khan et al. [52] suggested a deep neural network model based on RESNET-50 and RESNET-101 with a kurtosis-controlled principal component (KcPCA) approach. In contrast, Khan et al. [53] implemented a CAD system based on MASK-RCNN and a DenseNet deep model for lesion detection and recognition. Georgakopoulos et al. [54] compared two different CNN models without and with pretraining in images. The transfer learning technique was applied in the case of the pretrained model instead of randomly initialing the weights of the CNN. The consequences of this kind of hybrid method demonstrate that the classification results are significantly enhanced. Kulhalli et al. [55] provided a hierarchical classifier approach based on CNN and transfer learning. The proposed branched approach uses the pretrained InceptionV3 model for skin lesion classification. The structure of the hybrid methods based on transfer learning classifiers is presented in Figure 6.

### 3.3. Ensemble Methods

Tahir et al. [27] introduced a CNN-based method named DSCC_Net for the classification of skin cancer. ISIC 2020, DermIS, and HAM10000 were the three publicly accessible benchmark datasets utilized to evaluate the performance of the proposed methodology. Moreover, the performance of DSCC_Net was also compared with six baseline deep learning methods. Furthermore, the researchers used the SMOTE to effectively tackle the problem of underrepresented classes. The suggested DSCC_Net model showed a high level of effectiveness in accurately classifying the four distinct categories of skin cancer disorders. It achieved an impressive area under the curve (AUC) value of 99.43%, indicating its strong discriminatory power. Moreover, the model exhibited a commendable accuracy rate of 94.17%. The recall rate was found to be 93.76%, further highlighting the model’s reliability. The precision rate was determined to be 94.28%. Lastly, the F1-score, which combines precision and recall, was calculated to be 93.93%, further affirming the model’s overall performance in accurately classifying skin cancer disorders.

Karri et al. [56] developed a model by using two notable technical advancements: the evaluation of a two-stage, domain-to-domain transfer learning assessment, which involves model-level and data-level transfer learning that is carried out by fine-tuning two datasets, namely MoleMap and ImageNet. The authors introduced nSknRSUNet, a deep learning network designed for the segmentation of skin lesions. This network demonstrates good performance by using large receptive fields and feature fusion techniques to enhance spatial edge attention. The MoleMap and HAM10000 datasets were used to conduct a comparative analysis between the model’s predictions and images of real skin lesions originating from two separate clinical settings. The proposed model in data-level transfer learning, when applied to the HAM10000 dataset, attained a Dice Similarity Coefficient (DSC) of 94.63% and an accuracy of 99.12%. The MoleMap dataset demonstrated that the suggested model achieved a Dice Similarity Coefficient (DSC) of 93.63% and an accuracy of 97.01%.

Several research studies used ensemble methods, like Yu et al. [57], who proposed a network ensemble strategy to combine deep convolutional descriptors for automated skin lesion detection. In this proposed method, pretrained ResNet50 and VGGNet are adopted. Multiple CNNs are trained using a data augmentation technique specifically designed based on illuminant projection and color recalibration. Then, output convolution activation maps of each skin image are extracted from each network and the local deep features are selected from the object-relevant region. Finally, the Fisher kernel encoding-based method combines these deep features as image illustrations to classify lesions. SVM is then used to classify skin lesions accurately. Pal et al. [58] used an ensemble of three fine-tuned DenseNet-121, MobileNet-50, and ResNet50 architectures to predict the disease class.

Alizadeh et al. [59] proposed an ensemble method based on two CNN architectures, including a CNN model composed of nine layers and a pretrained VGG-19 CNN model combined with other classifiers. Milton [60] used an ensemble of transfer learning techniques including InceptionV4, SENet154, InceptionResNetV2, PNASNet-5-Large, and all architectures to classify seven different lesion classes. Chaturvedi et al. [61] implemented a method that uses five pretrained CNN models, including ResNetXt101, NASNet Large, InceptionResNetV2, InceptionV3, and Xception CNN, and four ensemble models to discover the best model for the multiclassification of skin cancer. However, Amirreza et al. [62] proposed a method that ensembles deep extracted features from several pretrained models.

Le et al. [63] provided an ensemble framework based on modified ResNet50 models with a focal loss function and class weight. Moreover, Mahbod et al. [64] described the effect of dermoscopic images of different resolutions on the classification performance of different fine-tuned CNNs in skin lesion analysis. Moreover, a novel fusion approach was presented by assembling the results of multiple fine-tuned networks, such as DenseNet-121, ResNet-50, and ResNet-18, that were trained with various dimensions and sizes of dermoscopic images. Nyiri and Kiss [65] suggested multiple novel methods of ensemble networks, such as VGG16, VGG19, ResNet50, Xception, InceptionV3, and DenseNet121, with differently preprocessed data and different hyperparameters to classify skin lesions. Bi et al. [66] implemented the CNN ensemble technique to classify nevi versus seborrheic keratosis versus melanoma from dermoscopic images; for this purpose, instead of training multiple CNNs, they trained three ResNet-like ResNet multiclasses for three classes; the second one is the other two lesion classes versus melanoma or the other two lesion classes versus seborrheic (ResNet binary) and for the third one, they ensembled the first two methods to obtain the final results (ResNet (ensemble)) by fine-tuning a pretrained CNN.

**Figure 7:**
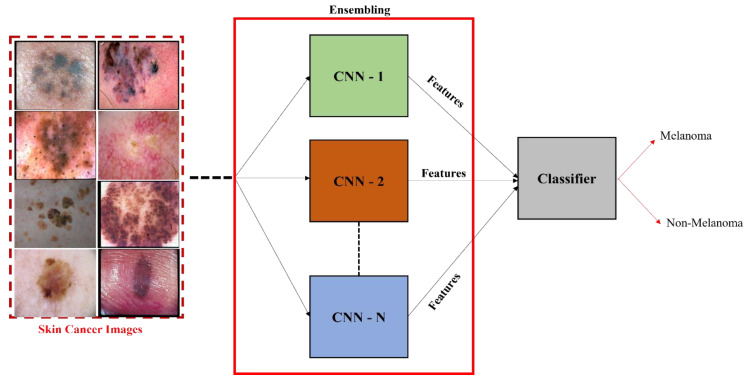
Ensemble CNN model for the classification of melanoma and nonmelanoma.

Wei et al. [67] proposed an ensemble lightweight melanoma recognition CNN model based on MobileNet and DenseNet. Harangi et al. [68] outlined that the ensemble of the different CNN networks enhanced the individual accuracies of models to classify different skin lesions into related classes such as seborrheic keratosis, melanoma, and benign. They fused the output layers of pretrained GoogLeNet, ResNet, AlexNet, and VGGNet CNN models. The best fusion-based methods were used to aggregate pretrained models into one framework. Finally, the extracted deep features were classified based on a sum of maximal probabilities. The overview of the ensembling of CNN-based models is depicted in Figure 7.

### 3.4. Federated Learning

**Figure 8:**
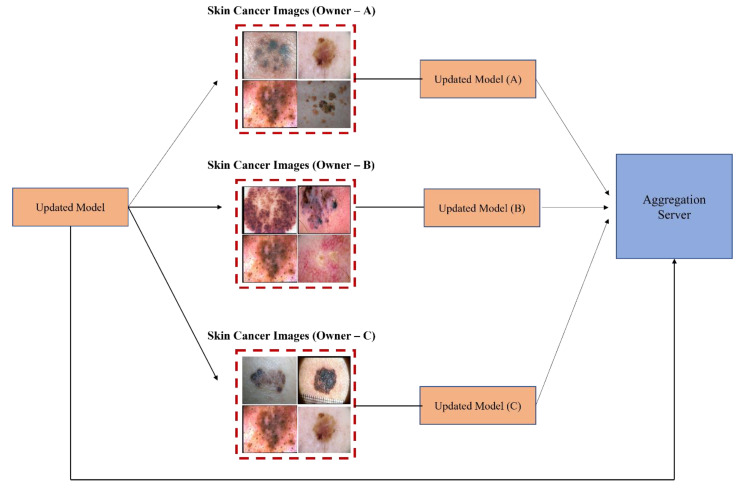
Federated learning for skin image classification.

Recently, federated learning (FL) has been proposed to train decentralized models in a privacy-preserved fashion depending on labeled data on the client side, which are usually not available and costly. To address this, Bdair et al. [31] proposed a federated learning method known as the FedPerl framework for classifying skin lesions using a multisource dataset. This study also applied various methods to improve privacy, hide user identity, and minimize communication costs. Lan et al. [69] proposed a MaOEA-IS based on federated learning to solve the privacy and fragmentation of data to a better extent for skin cancer diagnosis. Hossen et al. [70] applied federated learning based on a convolutional neural network to classify skin lesions using a custom skin lesion dataset while ensuring data security. Agbley et al. [71] proposed a federated learning model for melanoma detection that fused lesion images and their corresponding clinical metadata while ensuring privacy during training. Hashmani et al. [72] suggested an adaptive federated learning-based model with the capability of learning new features consisting of global point (server) and intelligent local edges (dermoscopy) that can correctly classify skin lesion dermoscopy images and predict their severity level. Moreover, Bdair et al. [73] proposed a semi-supervised FL framework motivated by a peer learning (PL) approach from educational psychology and ensemble averaging from committee machines for lesion classification in dermoscopic skin images. A block diagram of FL for the classification of skin lesion images is illustrated in Figure 8. In addition, Table 2 presents an overview of FL and transfer learning (TL) classifiers for skin disease classification.

## 4. Performance Evaluation of Methods to Determine the Efficacy of Various Classification Algorithms for Melanoma and Nonmelanoma Cancer Using Clinical and Dermoscopic Images (RQ2)

The classification accuracy of the considered articles was computed using evaluation metrics like TPR, TNR, PPV, ACC, and AUC. The credibility and performance of every classification method were judged on these metrics. The performance of the proposed models on single, multiple, and combined datasets was evaluated and a summary of the performance metrics is provided in Table 3.

### 4.1. Analyzing Performance on a Single Dataset

Damian et al. [79] proposed a CAD method based on texture, color, shape, and deep learning feature fusion through mutual information (MI) metrics for nonmelanoma and melanoma lesion detection and classification. The efficiency of this method was tested on the HAM10000 dataset and exhibited competitive performance against other advanced methods. Furthermore, Adegun and Viriri [80] implemented a CAD framework based on a segmentation network with a multi-scale encoder–decoder and a fully convolutional network-based DenseNet classification network combined with a fully connected (FC) CRF for the refinement of skin lesion borders to generate precise soft segmentation maps, as well as a DenseNet architecture for the effective classification of lesion images. Furthermore, Nida et al. [81] implemented a deep regional CNN with FCM clustering for skin lesion segmentation and detection. Moreover, Kaymak et al. [82] utilized four different FCNs, namely FCN-8s, FCN-16s, FCN-32s, and FCN-AlexNet, for the automatic semantic segmentation of lesion images. Shan et al. [83] implemented an ∇N-Net architecture with a feature fusion method; all these proposed methods were tested and evaluated on the ISIC 2018 benchmark dataset. Bakheet et al. [84] proposed a CAD method based on multilevel neural networks with improved backpropagation based on the Levenberg–Marquardt (LM) model and Gabor-based entropic features. Balaji et al. [85] implemented a firefly and FCM-based neural network. The performance of the classification methods was evaluated on an open-source PH2 dataset that consists of only 200 lesion images, including 40 melanoma images, 80 atypical nevi, and 80 common nevi images. Warsi et al. [86] proposed a novel multi-direction 3D CTF method for the extraction of features from images and employed a multilayer backpropagation NN technique for classification.

### 4.2. Performance Evaluation on Multiple Datasets

Yutong Xie et al. [87] proposed a mutual bootstrapping DCNN method based on coarse-SN, mask-CN, and enhanced-SN for simultaneous lesion image segmentation and classification, and the effectiveness of the proposed approach was validated using ISIC 2017 and PH2 datasets. Barata et al. [88] proposed a multi-task CNN with channel and spatial attention modules that perform a hierarchical diagnosis of the lesion’s images and used ISIC 2017 and ISIC 2018 datasets to evaluate the proposed model. Hosny et al. [89] implemented a method that used ROI and data augmentation techniques and modified GoogleNet, Resnet101, and Alex-Net models. The performance and effectiveness of the proposed approach were validated using ISIC 2017, DermIS, DermQuest, and MED-NODE datasets. Filali et al. [90] used PH2 and ISIC 2017 to validate a method based on the fusion of features like texture, color, skeleton, shape, and four pretrained CNN features. Moreover, Hasan et al. [91] proposed a lightweight DSNet that uses PH2 and ISIC 2017. Saba et al. [92] used PH2, ISIC 2017, and ISIC 2016 benchmark datasets to evaluate their proposed contrast-enhanced deep CNN method. The deep features were extracted through AP and FC layers of the pretrained Inception V3 algorithm through fine-tuning.

### 4.3. Performance Evaluation on Combined Datasets

Javeria et al. [93] implemented an architecture that extracts deep features using AlexNet and the VGG-16 model and fused them to produce a single feature vector for melanoma classification. Optimal features were selected by using the PCA method. This model was assessed on a combined dataset which contains 7849 images of the ISIC 2016, ISIC 2017, and PH2 datasets. Hameed et al. [94] implemented a method based on AlexNet for performing multiclass, multilevel classification. The pretrained model AlexNet was re-trained on the multisource dataset by performing fine-tuning. The proposed method was validated using 3672 images gathered from different online data sources such as DermQuest, DermIS, DermNZ, ISIC Image Challenge, and PH2. Zhang et al. [95] proposed an optimized CNN technique that adopted a whale optimization method for improving the efficacy of CNNs and evaluated the method on a large DermIS and DermQuest combined dataset. The proposed method was compared with other pretrained CNNs and gave the best results for melanoma diagnosis.

### 4.4. Performance Evaluation on a Smartphone Camera-Based Collected Dataset

Pacheco and Krohling [96] suggested an aggregation method combining patient clinical information with pretrained models. To validate the proposed method, the PAD dataset was used based on the images collected by using different smartphone cameras. The model achieved an improvement of approximately 7% in balanced prediction accuracy.

**Table 3:** Performance evaluation of TL and FL classifier.

Ref.	Dataset	Model	TPR	TNR	PPV	ACC	AUC
[18]	ISIC 2019	SLDCNet, FrCN	99%,	99.36%	NM	99.92%	NM
[69]	ISIC 2018	MaOEA-IS with FL	NM	NM	NM	91%	88.7%
[87]	ISIC 2018	CKDNet	96.7%	90.4%	NM	93.4%	NM
[88]	ISIC 2017	CKDNet	92.5%	70%	NM	88.1%	90.5%
[97]	ISIC 2019	GoogleNet and transfer learning	79.8%	97%	80.3%	94.92%	NM
[98]	ISIC 2019	ResNet-101, NASNet-Large	88.46%	88.24%	NM	88.33%	NM
[99]	ISIC 2019	Adaptive ensemble CNN with FL	91%	NM	90%	89%	NM
[100]	ISIC 2018	Ensemble GoogLeNet, Inceptionv3	45%	97.2%	67.5%	88.2%	91.3%
[101]	ISIC 2018	∇N-Net architecture	NM	NM	NM	87%	NM
[102]	ISIC 2018	Hybrid-CNN with DSNet	86%	NM	85%	NM	97%
[103]	ISIC 2017	FrCN	78.9%	96%	NM	90.7%	NM
[104]	ISIC 2017	Mutual bootstrapping DCNN	72.7%	91.5%	NM	87.8%	90.3%
[105]	ISIC 2017	Ensemble CNN	NM	NM	NM	NM	92.1%
[106]	ISIC 2017	Inception-V3	94.5%	98%	95%	94.8%	98%
[107]	ISIC 2017	DenseNet-161, ResNet-50	60.7%	89.7%	NM	NM	80.0%
[108]	ISIC 2017	FC-DenseNet	83.8%	98.6%	NM	93.71%	NM
[109]	ISIC 2017	Lightweight DSNet	83.6%	93.9%	NM	92.8%	NM

## 5. Available Datasets for the Evaluation of Classification Methods for Melanoma and Nonmelanoma Skin Cancer (RQ3)

**Figure 9:**
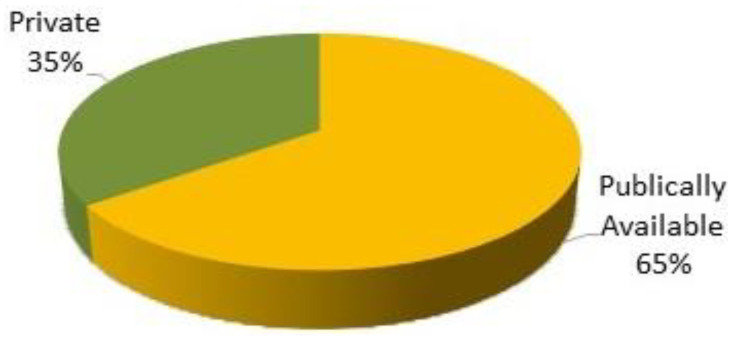
Availability proportion of datasets.

There were numerous datasets available for skin lesion classification. It was identified from the literature that most datasets are publicly available for use, and some are not publicly accessible. Figure 9 shows the availability proportion of public and private datasets.

### 5.1. Public Datasets

These datasets are also known as benchmark datasets because of their high usage in research for detecting melanoma. The below-discussed datasets are known as benchmark datasets.

SIIM-ISIC 2020 challenge dataset: This dataset contains 33,126 dermoscopic images of different types including 584 melanoma and nonmelanoma images [29]. These images were collected at multiple centers and are available in two formats, DICOM/JPEG and TIF. This multicenter dataset was collected from 2056 patients worldwide with clinical contextual information.

ISIC 2019 challenge dataset: This dataset comprises 25,331 dermoscopic images of eight types and includes 4522 melanoma images, with the rest being nonmelanoma images [39,106].

ISIC 2018 challenge dataset: This dataset consists of 12,500 dermoscopic images of seven types such as dermatofibromas, vascular lesions, Bowen’s disease, actinic keratosis, BCC, seborrheic keratosis, nevi, and melanoma [110].

ISIC 2017 challenge dataset: This dataset contains 2000 dermoscopic images of three types, of which 374 are melanoma, 1372 are benign nevi, and 254 are seborrheic keratosis [99,101].

ISIC 2016 challenge dataset: This dataset has a collection of 900 images including 173 melanoma and 727 noncancerous, labeled as either malignant or benign [82].

PH2 dataset: This dermoscopic image database consists of 200 images, which contain 40 melanoma, 80 atypical nevi, and 80 common nevi, obtained from the “Pedro Hispano Clinic, Portugal Dermatology Service” [76,83,92].

HAM10000 dataset: This is a benchmark dataset with a massive collection of multisource dermoscopic lesion images extracted from the ISIC 2018 grand challenge datasets. The dataset contains 10,015 images of seven different types of common pigmented skin lesions, namely MEL, VASC, AKIEC, NV, BCC, DF, and BKL, with a 600 × 450-pixel resolution including 1113 melanoma images [78,95,97].

MEDNODE dataset: This dataset has a collection of 170 non-dermoscopic images of two types, including 100 nevus images and 70 melanomas from the digital image archive of the University of Medical Center’s Department of Dermatology, Groningen (UMCG). The dimensions of clinical images range from 201 × 257 pixels to 3177 × 1333 pixels [111].

DermIS: The DermIS Digital Database is a European dermatology atlas for healthcare professionals. This image database consists of 206 images of different types including 87 benign and 119 melanoma images in RGB format. It has vast online lesion image information for detecting skin cancers on the Internet [112]. The images in the datasets consist of two labeled classes, nevus and melanoma. This organization provides free-to-use classified images for academic purposes [113].

ISIC Archive: This online archive dataset has a collection of around 24,000 clinical and dermoscopic high-quality images of seven different types, including 2177 melanoma images. Their growing archive is labeled manually, containing high-quality lesion images [114].

DERM 7pt: This dataset consists of a seven-point skin lesion malignancy checklist. It comprises 1011 images, including 252 melanoma and 759 benign [115].

DermNet: The DermNet dataset is freely available, gathered and labeled by the DermNet Skin Disease Atlas, and has a collection of around 23,000 dermoscopic images, of which around 635 are melanoma. This dataset consists of 23 super-classes and 642 sub-classes of the disease [86,107,116].

DermQuest: The DermQuest database is an online medical atlas for educationists and dermatologists. It provides 22,000 non-dermoscopic (clinical) images for analysis purposes. Renowned international editorial boards reviewed and approved these clinical images. The images in the datasets have only two labeled classes, nevus and melanoma. These organizations provide free-to-use classified images for academic purposes [116,117].

### 5.2. Private Datasets

DermNet NZ: The dermatology image library owned by the DermNet New Zealand Trust contains over 20,000 clinical images for download and re-use [118]. It is frequently updated to provide information about the skin via any desktop or mobile web browser. Moreover, high-definition, non-watermarked images are available for purchase [108,117,119].

Dermofit Image Library: This dermoscopic image database contains 1,300 high-quality images including 526 melanomas. The Dermofit dataset consists of 10 different classes of lesions, such as melanocytic nevus (mole), actinic keratosis, intraepithelial carcinoma, basal cell carcinoma, pyogenic granuloma, seborrheic keratosis, hemangioma, pyogenic granuloma, dermatofibroma, and squamous cell carcinoma [120]. A licensing agreement with a EUR 75 license fee is required to obtain this dataset [90,101].

Interactive Dermoscopy Atlas: The Interactive Dermatology Atlas database consists of over 1000 clinical and dermoscopic images from patient visits over the past two decades [121]. This dataset contains 270 melanomas, 681 unmarked, and 49 seborrheic keratosis. This database is accessible by paying a fee of EUR 250 for research purposes [84,92].

### 5.3. Non-Listed/Non-Published Datasets

MoleMap Dataset: This is a New Zealand-forward telemedicine service and store for diagnosing melanoma. It contains 32,195 photographs of 8882 patients with 14,754 lesions from 15 disease groups and it was collected between the years 2003 and 2015. Clinical and dermoscopic images of skin lesions are included in the dataset and image size varies from 800 × 600 pixels to 1600 × 1200 pixels [122]. This dataset is available only upon special request [77,88].

Irma skin lesion dataset: This dataset comprises 747 dermoscopic images, including 560 benign and 187 melanoma lesions, with a resolution of 512 × 512 pixels. It is under third-party development and only available upon special request [88,120].

Skin-EDRA. The Skin-EDRA dataset consists of 1064 clinical and dermoscopic images with a 768 × 512-pixel resolution. This dataset is a part of the EDRA CDROM collected as a CD resource from two European university hospitals [66,77,123].

PAD dataset: The Federal University of Espírito Santo collected this dataset through the Dermatological Assistance Program (PAD) [124]. This dataset consists of 1612 images collected using various smartphone camera devices with different resolutions and sizes. Their respective clinical information includes the patient’s age, lesion location on the body, and whether the lesion has recently increased, changed its pattern, itches, or bleeds. Moreover, the dataset contains six types of skin lesion images (MEL 67, ACK 543, BCC 442, NEV 196, SCC 149, and SEK 215) and is available only upon special request [57,60].

It was identified from the literature that publicly available datasets were the most preferred datasets until February 2023 and were most frequently used by researchers to evaluate their proposed architectures. 

**Figure 10:**
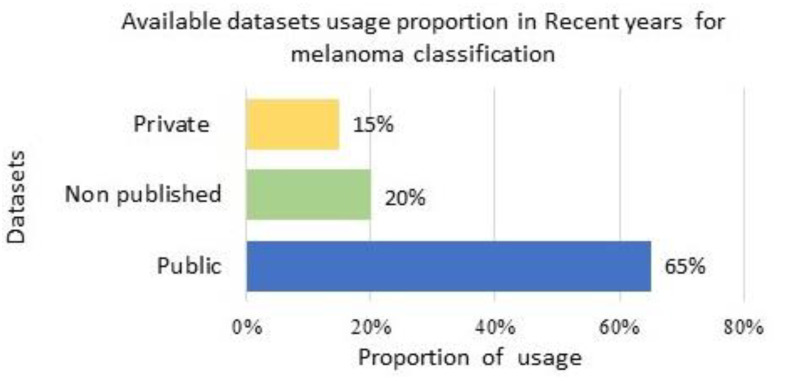
Dataset usage proportion for method evaluation: usage proportion of available public, non-listed, and private datasets in current years for melanoma classification.

Figure 10 presents the recent trend in using available public, private, and non-listed datasets for melanoma classification.

## 6. Taxonomy for Melanoma Diagnosis

**Figure 11:**
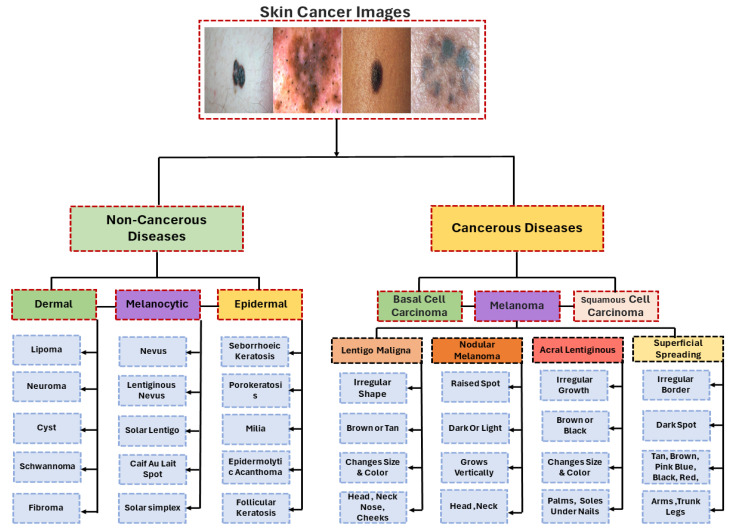
Taxonomy for melanoma diagnosis.

Initially, the proposed taxonomy classifies lesion images into cancerous diseases (malignant) and noncancerous diseases (benign) [125]. In this study, the majority of the studies examined were on transfer learning for the categorization of melanoma and nonmelanoma skin cancers. Squamous cell carcinoma and basal cell carcinoma are considered nonmelanoma cancers [126,127]. Melanoma is the most serious kind of skin cancer. Lentigo maligna, acral lentiginous, noda melanoma, and superficial spreading are the four main subtypes of melanoma. Malignant melanoma is the name given to these cancers when they are found. Finding the appropriate form requires analysis and searching for patterns. A model is trained to identify the particular type of cancer [62,66,77,78]. There is a wide range of melanoma cancers, each with its own unique appearance, size, structure, prevalence, and color. Lentigo maligna has an uneven form that may be brown or tan and varies in color and size, while Noda melanoma has elevated patches that can be dark or light and develop vertically. Acral lentiginous melanoma grows unevenly and varies in size and color, while superficial spreading melanoma is characterized by a black patch, an uneven border, and color variations. Additionally, if the illness is determined to be benign or noncancerous, it is divided into three primary categories: dermal, epidermal, or melanocytic [62,78]. These skin cancers have shapes that look like melanoma. They are not cancerous and belong to the group of noncancerous diseases (Figure 11).

Several techniques such as precision, recall, and F1-score are used to analyze the performance of methods. The precise measurement of classification performance is provided by considering all values in the confusion matrix. The Matthews Correlation Coefficient (MCC) is a statistical measure that assume values between −1 and 1. A value of −1 represents complete disagreement, while a value of 0 suggests no improvement compared to random classification. The metric under consideration is a quantitative assessment of the effectiveness of categorization, accounting for the potential occurrence of chance results. A value of 1 represents complete consensus, whereas 0 signifies no discernible enhancement above random chance and −1 denotes a lack of consensus. The range of Kappa values spans from −1 to 1. The distribution of accurate replies is determined by the percentage of correct, incorrect, and incomplete responses. The Jaccard index is a metric used to evaluate the performance of a model by comparing the agreement between its predicted outcomes and the precision of manually annotated examples.

However, the MCC measure has many benefits in comparison to other metrics, including precision, confusion entropy, F1-score, and balanced precision. The great reliability of the Matthews Correlation Coefficient (MCC) with imbalanced databases is attributed to its ability to provide a high score when a significant proportion of both projected negative and positive data occurrences are accurately classified.

## 7. Results and Discussion

Our systematic review study included 86 research papers in the domain of FL and transfer learning methods for melanoma and nonmelanoma skin cancer detection. Different transfer learning-based training methods and algorithms were employed across research studies for diagnosing melanoma and nonmelanoma from dermoscopic and non-dermoscopic images. The most commonly used pretrained models were ResNet-50, DenseNet, VGG-16, VGG-19, MobileNet, Inception V3, Xception, GoogleNet, and AlexNet [68]. Several effective frameworks and architectures based on transfer learning have been suggested by researchers in recent years [62,66,74,76,83]. The effectiveness of these methods was discerned and observed in melanoma detection from clinical and dermoscopic images across selected studies. Some proposed methodologies use an ensemble of several pretrained models and aggregate their predictions to “boost” model performance. The application of ensembling can be astonishingly beneficial, not only for integrating multiple pretrained models but also for merging distinct hyperparameter choices for these networks.

Some researchers suggested an integrated two-phase framework based on FCN methods for segmenting and classifying multiple skin lesions [63]. The pre-segmentation phase enables these classifiers to memorize and learn only specific skin lesion features while ignoring the surrounding region and giving a better segmentation output. Many researchers implemented hybrid methods in which different preprocessing and data augmentation techniques like flipping, crop, zoom, and rotate operations were performed on a skin color image dataset for the augmentation of segmented ROI images to solve the problem of overfitting and imbalanced datasets; in addition, various fine-tuned pretrained models were employed to extricate deep features from lesion images to diagnose lesions as melanoma or nonmelanoma [76]. Some researchers suggested federated learning-based methods for melanoma and nonmelanoma skin cancer diagnosis to resolve the issue of the small number of lesion images in a dataset for the training of the model without compromising user-sensitive data. All these proposed methods were tested and evaluated on various public benchmark datasets as well as non-public, combined, and non-listed datasets. It was seen that the validation methods’ effectiveness and efficacy fluctuated and different results were obtained among various research studies [77,79]. These selected research studies provided sufficient data to construct evaluation tables to calculate performance metrics. It was observed from the literature that the FCN-based method’s accuracy ranged from approximately 81% to 98%, and the accuracy of ensemble and hybrid methods ranged from approximately 76% to 99%. In comparison, federated learning-based methods ranged from approximately 81% to 91% when evaluated on various datasets, including open access, private, and combined datasets [89,90]. Research in the domain of diagnosing skin cancer is making encouraging progress. Regardless of the fact that now is an auspicious and providential moment for approaches based on transfer learning, it was observed from the literature that there are undeniable challenges and problems faced by these approaches in becoming perfect and effective diagnostic methods, which one needs to resolve in imminent stages. Now, researchers claimed that their proposed model beats doctors’ performance in the classification of melanoma and nonmelanoma skin cancer. Still, this view is far from reality because they experimented in a closed environment with defined principles. These systems are never tested on cancer patients in real-life diagnosis [84,90]. The real-world diagnosis process needs to consider the patient’s ethnicity, existing sun damage, eye color, hair, skin, medicines, the number of nevi, occupation, illness, response to previous therapy, treatments, and lifestyle habits like alcohol intake, smoking, sun exposure, clinical history, and other data from the patient’s medical records. There are many inter-class dissimilarities and intra-class similarities concerning size, color, place, and texture in the visual appearance of lesions. But despite this, current algorithms and approaches based on transfer learning conspicuously depend only on patients’ imaging data [116,117]. However, when these systems are employed for skin lesion images, they might have a greater risk of misdiagnosis. It was observed from the literature that transfer learning algorithms need a substantial and extensive amount of high-quality, balanced, and diverse training data that indicate each class of skin lesions to revamp the recognition accuracy of methods. Federated and transfer learning-based systems have the potential to bring out a progressive change in the detection of melanoma and nonmelanoma skin cancer and enable a remotely accessible, affordable, and cost-effective procedure [109]. To improve existing AI systems and enhance the classification accuracy of methods, dermatologists and computer vision societies need to work and collaborate.

## 8. Research Gap and Future Direction for Melanoma and Nonmelanoma Skin Cancer Classification

The research gap and corresponding future directions for diagnosing melanoma and nonmelanoma skin are presented in this section. State-of-the-art classification methods are facing numerous challenges that can be identified in the selected articles.

### 8.1. Challenges in Transfer Learning-Based Classification Methods

This section describes the six significant challenges that were identified in the literature.

#### 8.1.1. Dataset Inconsistency

The datasets were collected from heterogeneous sources and, hence, have many inconsistencies. In the DermIS and DermQuest online data repositories, various people have uploaded their images of skin lesions, but their image-capturing devices are different. Hence, for each image, the dimension, type, quality, and format of images may fluctuate. In these datasets, shape and boundary features are not extracted accurately because some of the images cover the lesion area. In contrast, most of the images cover the whole body, which restricts the feature extraction stage in conventional approaches [99,107]. Thus, the poor resolution of these images may affect classification performance. So, the inconsistency of the dataset is a considerable challenge.

#### 8.1.2. The Lack of Lesion Images from Dark-Skinned People in the Datasets

Typically, current datasets consist of lesion images that belong to only white and fair-skinned people rather than dark-skinned or brown people. Every year since 2016, ISIC has announced a challenge to tackle melanoma detection, but the limitation of this ISIC dataset is that it has image data from mostly fair-skinned people [104,107]. Dark- or brown-skinned people can also have cancer and are usually detected at later stages. Hence, deep and transfer learning architectures that are tested and validated for detecting melanoma in light-skinned people have a higher risk of misdiagnosing those with brown or darker skin.

#### 8.1.3. ABCDE Rule of Dermoscopy

In the clinical environment, the dermoscopy technique is used to visually examine suspected skin lesions. The ABCDE rule is a fundamental constraint for distinguishing between benign and malignant lesions. The ABCDE rule comprises whether the skin lesion is asymmetrical, has irregular borders, shows different colors, whether its diameter is larger than 6 mm, and whether the color of the lesion has changed. Hence, deep learning- and transfer learning-based models do not perform as well as the ABCDE rule, which is trusted by dermatologists. The main reason is the pattern recognition complexity for malignant lesion characteristics in medical imaging. That is why recent attempts are still considered black-box approaches [62,98].

#### 8.1.4. The Limited Number of Images in Datasets

It was observed that publicly available datasets consist of small lesions for training and testing. The proposed models’ performance is good on a limited number of images, while their credibility is unpredictable when these models are tested on a vast image set [93,95,121].

#### 8.1.5. Patient’s Clinical Metadata and Case History

Patient’s case history and clinical metadata, such as age, sex, structure, lesion size, ethnicity, and patient family history of skin cancer, are considered very significant when carrying out a visual examination of a suspicious lesion through dermoscopy. Therefore, image-based deep and transfer learning methods suggested for the classification of melanoma falter for crucial characteristics of patient and clinical metadata [79]. Moreover, it was observed that in most available datasets, both patient history and clinical metadata are unfortunately missing.

#### 8.1.6. Unbalanced Datasets

In different available datasets, it is commonly seen that there are primarily images of benign skin lesions rather than malignant lesions. There is a scarcity of rare lesions such as vascular, dermatofibroma, and actinic keratosis, not only in the ISIC 2018 dataset but in all publicly available datasets. Most deep learning- and transfer learning-based methods are trained on a balanced dataset. Hence, the performance of algorithms is usually affected by insufficient and unbalanced datasets [122].

### 8.2. Potential Future Opportunities and Work

This section describes the possible future opportunities that should be considered to improve the performance of AI-based systems. Deep learning- and transfer learning-based approaches could improve the detection of skin cancer with the opportunities specified below.

#### 8.2.1. Miscellaneous Datasets

In datasets, included skin lesion images are mostly of fair-skinned people. Datasets must have racial and ethnic miscellany and diversity; they must include equally distributed dark-skinned and fair-skinned lesion images to minimize ethnic or social bias in frameworks. The very same consideration can be diversified or enhanced for age, particularly when surrounding solar damage or degree of skin aging, which can influence decision making and the dataset [68,75,77].

#### 8.2.2. Generative Adversarial Networks

Currently, GANs are mainly used to create high-resolution fake image data to manage the problem of small and limited datasets. For melanoma and nonmelanoma skin cancer, GANs produce realistic synthetic images to overcome the insufficiency of annotated data. For synthesizing fine-grained and good-quality lesion images, Abdelhalim et al. [23] suggested a self-attention-based progressive GAN (SPGGAN). The distribution of skin lesions is heavily distorted or biased in publicly available datasets. GANs can be utilized to produce lesion image data for rare classes of skin cancer, namely carcinoma, Kaposi sarcoma, sebaceous, and MCC.

#### 8.2.3. Data Fusion Algorithm Development

Data fusion algorithms need to be developed to integrate the features of images from deep learning models with patient clinical information to provide a final output for the diagnosis of melanoma and nonmelanoma skin cancer, because patient history and clinical metadata have significant importance in the diagnosis of skin cancer [16].

#### 8.2.4. Federated Learning-Based Framework Development

The issue of the limited no. of lesion images in a dataset for the training of models has been resolved by FL without compromising the privacy of user information. So, more frameworks should be developed based on FL in the future for melanoma and nonmelanoma skin cancer diagnosis [28,35,37,123].

#### 8.2.5. Data Augmentation Techniques

Data augmentation techniques could improve the detection of melanoma and nonmelanoma skin cancer. The addition of augmented samples with various image transformation techniques, such as vertical and horizontal flip, color jitter, color space, translation, rotation at different angles, and random crop, may reduce many limitations of skin lesion datasets such as heterogeneous sources of image data and unbalanced data between the classes of lesions [23].

#### 8.2.6. Color Constancy Algorithm Development

Skin lesion images are obtained from various image-capturing devices with different illumination settings in publicly available dermoscopic and clinical datasets, which could decrease the performance of the deep learning and transfer learning models [124]. It has been proved through many types of research that color constancy methods like max-RGB and Shades of Gray can be utilized to enhance the efficiency of pretrained models for the classification of heterogeneous data source images [30,41,71].

#### 8.2.7. A Balanced Skin Lesion Dataset

A balanced skin lesion dataset is essential to achieve superior performance with transfer learning algorithms; hence, the selection of cases with balanced datasets is a requisite that would perfectly represent the class of a specific lesion, and the input of proficient doctors could be beneficial, productive, and worthwhile for this selection [38,41].

#### 8.2.8. CAD System Design Based on the ABCDE Medical Algorithm and Transfer Learning

The ABCDE rule is considered a significant constraint for distinguishing between benign and suspicious malignant skin lesions. Hence, a deep and transfer learning automatic diagnostic system based on the commonly well-proven ABCDE medical procedure can be developed that performs in the same way as the ABCDE rule, trusted by dermatologists, in order to enhance the performance of pretrained models [71].

#### 8.2.9. Internet of Things (IoT) and Transfer Learning

Cloud computational power and storage are becoming more cost-effective and affordable [125,128]. So, a fast, automatic, and accessible system can be designed by using the concept of the Internet of Things (IoT) and transfer learning in parallel to assist dermatologists with skin lesion diagnoses in clinical cases around the world [97,123].

### 8.3. Limitations

In this SLR, we followed Kitchenham’s approach for performing systematic reviews [28] to avoid selection bias instead of depending only on our information and background. In total, 86 articles in English met the inclusion criteria and qualified for further assessment. Thus, it is possible that articles in other languages and related gray literature were missed. We performed a search operation by using many keywords and terms related to melanoma skin cancer because researchers from different backgrounds use different terms for the same concept and topic. Our search was concluded in February 2023, so research studies published after that date would not have been taken into consideration or captured. Aside from these limitations, to the best of our knowledge, this is the first SLR on diagnosing melanoma through CNN-based pretrained models and federated learning and it could be helpful for other researchers to plan their research activities.

## 9. Conclusions

This systematic review study discussed the latest research on melanoma and nonmelanoma skin cancer classification using federated and transfer learning techniques. This SLR was designed to provide contemporary research on the performance and effectiveness of transfer learning- and federated learning-based models used for detecting melanoma and nonmelanoma skin cancer across several modalities of skin lesion datasets. In this review, various transfer learning- and federated learning-based approaches and classification methods to diagnose melanoma and nonmelanoma skin cancer were analyzed extensively, and we also highlighted the principal shortcomings of existing approaches and areas of research where further enhancement should soon be carried out. Moreover, various skin lesion datasets that are publicly available, as well as private and non-listed ones, including dermoscopy, whole-slide pathology scanning (histopathology images), and clinical images, were described. Furthermore, a taxonomy was proposed by exploring relevant research studies. Moreover, the research gap and future direction of AI-based systems were subsequently described and established in this SLR. There were six existing issues of classification systems identified and nine potential opportunities were suggested to resolve the identified challenges and enhance the performance of federated learning- and transfer learning-based systems, so that they can be used as a powerful aid for dermatologists and their performance in diagnosing skin cancer can be enhanced. In the future, researchers must perform an analysis on the graph and signal processing techniques for detecting melanoma and nonmelanoma skin cancers.

## Figures and Tables

**Table 1:** Research questions (RQs).

No.	Research Question	Motivation
RQ1	What types of the best available methods are used for the detection of melanoma and nonmelanoma skin cancers from clinical and dermoscopic images?	To explore different types of transfer learning- and federated learning-based approaches that are used for melanoma and nonmelanoma skin cancer diagnosis from clinical and dermoscopic images.
RQ2	What types of metrics are used to determine the efficacy of various classification algorithms for melanoma and nonmelanoma skin cancer diagnosis from clinical and dermoscopic images?	To identify the performance metrics of federated- and transfer learning-based algorithms like true positive rate (TPR), true negative rate (TNR), precision (PPV), accuracy (ACC), and area under the curve (AUC).
RQ3	What types of datasets are available for the detection of melanoma and non-melanoma skin cancer? What is the credibility and reliability of these datasets?	To explore the availability of publicly available datasets as well as non-listed, private datasets.

**Table 2:** Federated and transfer learning classifiers for the classification of melanoma.

Ref	Training Algorithms	Archi.	Datasets	Image Modality
[17]	Hybrid deep CNN	DCNN	HAM10000, ISIC 2018	Dermoscopy
[18]	SLDCNet, FrCN	DCNN	ISIC 2019	Dermoscopy
[31]	FedPerl	FL	Multisource combined dataset	Dermoscopy
[42]	MaOEA-IS (federated learning)	FL	ISIC 2018	Dermoscopy
[44]	AlexNet + LDA	CNN	ISIC Archive	Dermoscopy
[45]	ResNet-18, VGG16, AlexNet	DNN	ISIC 2016, ISIC 2017	Dermoscopy
[47]	LeNet + Adaptive linear piecewise function	CNN	ISIC 2018	Dermoscopy
[48]	AlexNet	DNN	PH2	Dermoscopy
[66]	DenseNet	DCNN	ISIC 2017, HAM10000	Dermoscopy
[67]	MobileNet V1, DenseNet-121	DCNN	ISIC 2016	Dermoscopy
[68]	CNN	DCNN	Dermo fit, MEDNODE	Dermoscopy
[69]	MaOEA	FSDM	Ham 10000	Dermoscopy
[70]	FL + CNN	CNN	Custom image dataset	Dermoscopy
[71]	FL + CNN	FL	Multisource dataset	Dermoscopy
[72]	Adaptive ensemble CNN with FL	FL	ISIC 2019	Dermoscopy
[74]	Ensemble DCCN	DCNN	ISIC 2017, PH2	Dermoscopy
[75]	Derma Net	CNN	ISIC 2017	Dermoscopy
[76]	VGG-M, VGG-16	DNN	ISIC 2016, Atlas	Dermoscopy
[77]	Ensemble CNN	CNN	HAM 10000	Dermoscopy
[78]	CNN	CNN	ISIC 2017, ISIC 2016, PH2	Dermoscopy

## Data Availability

Not applicable.
